# The role of radiotherapy in patients with advanced melanoma failing targeted therapy

**DOI:** 10.2340/ao.v65.45437

**Published:** 2026-04-24

**Authors:** Ellen Heurlin, Lina Grödeberg, Hildur Helgadottir

**Affiliations:** a Department of Oncology-Pathology, Karolinska Institutet, Stockholm, Sweden; b Theme Cancer, Karolinska University Hospital, Stockholm, Sweden

**Keywords:** melanoma, radiotherapy, molecular targeted therapy, drug resistance, proto-oncogene proteins B-raf

## Abstract

**Background and purpose:**

Approximately 50% of patients with metastatic melanoma harbor mutations in the *BRAF* gene, making them eligible for targeted therapy (TT). However, treatment options become limited once resistance develops. The role of radiotherapy (RT) in this context remains uncertain, and concerns exist regarding toxicity when RT is delivered concurrently with TT.

**Patient/material and methods:**

This retrospective study included metastatic melanoma patients treated with RT after experiencing disease progressing on TT between 2015 and 2023. Patients were grouped by subsequent systemic therapy: RT-STOP (discontinued TT), RT-TT (continued TT), and RT-ICI (switched to immune checkpoint inhibitors (ICI)). Study endpoints were progression-free survival (PFS), overall survival (OS), efficacy, and toxicity.

**Results:**

Sixty-three patients were analyzed. Median PFS and OS were 1.9 and 3.1 months. The median OS in RT-STOP, RT-TT, and RT-ICI was 1.7, 4.7, and 3.0 months, while the 1-year OS rate was 4.9, 7.6, and 33.4%, respectively (*p* = 0.001). RT was well tolerated, with no grade ≥3 adverse events observed and 50.9% of patients derived a local benefit.

**Interpretation:**

In advanced melanoma patients with disease progression on TT, RT was safe and provided a local effect. Although survival outcomes remained suboptimal, continuation of TT beyond progression or transition to ICI following RT was associated with improved OS compared with discontinuation of TT. These results support the role of RT as a safe bridging modality, suggest benefit from treatment beyond progression with TT, and warrant confirmation in prospective trials.

## Graphical abstract

**Figure:**
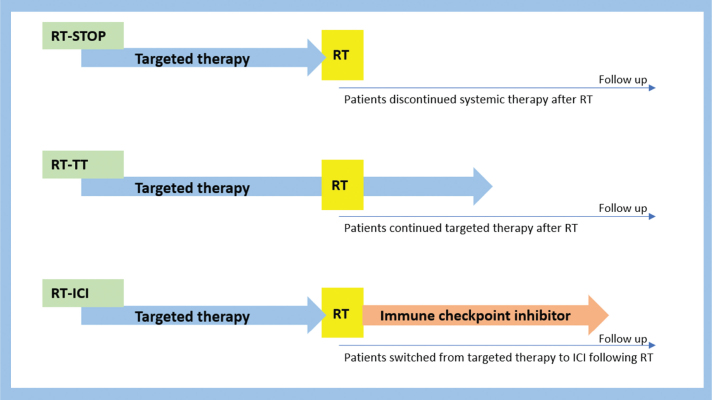

## Introduction

Approximately half of all patients with cutaneous melanoma have activating tumor mutations in the *BRAF* gene, which is part of the mitogen-activated protein kinase (MAPK) signaling cascade that regulates tumor growth, differentiation, and apoptosis [1]. *BRAF* mutations, commonly in the V600 locus, occur more frequently in younger patients and in tumors arising on non-chronically sun-exposed skin [2]. In metastatic *BRAF* V600 mutated melanoma, the advent of targeted therapies (TT) has markedly improved survival, with the U.S. Food and Drug Administration (FDA) and European Medicines Agency (EMA) approval of the BRAF inhibitor (BRAFi) vemurafenib in 2011 [3] and the MEK inhibitor (MEKi) trametinib in 2013 [4]. Currently, approved BRAFi include vemurafenib, dabrafenib, and encorafenib, in combination with the MEKi cobimetinib, trametinib, and binimetinib, respectively [5–7].

Despite the initially potent antitumor effect often achieved with TT, therapeutic resistance inevitably develops. Reported median progression-free survival (PFS) in advanced *BRAF* V600 mutated melanoma is 7–9 months with BRAFi monotherapy and 11–15 months with combination BRAFi/MEKi [8–10]. Since 2023, results from the DREAMseq and SECOMBIT trials have suggested that BRAFi/MEKi should be used as second-line treatment following immune checkpoint inhibitors (ICI), unless the patient presents with aggressive and symptomatic disease. Furthermore, these studies have demonstrated that the likelihood of response to ICI after progression on BRAFi/MEKi is lower compared with patients naive to ICI [11, 12]. Managing disease progression with BRAFi/MEKi remains a clinical dilemma due to limited treatment options, especially in patients who have also failed ICI. Although survival outcomes in this setting are less reported, studies indicate that survival is poor [11, 13–15]. In this context, the efficacy of radiotherapy (RT) and optimal management with subsequent systemic treatments has not been well studied. Additionally, given the limited prognosis, it is essential to determine whether patients gain a clinically meaningful benefit from RT. Furthermore, concerns have been raised regarding potential toxicity when RT is administered concurrently with TT. Early BRAF/MEKi trials prohibited concomitant RT following regulatory safety concerns about enhanced radiation toxicity [5–7]. As safety data accumulated, the later COLUMBUS trial recommended pausing TT for five drug half-lives before and after RT – equivalent to 1 day for encorafenib monotherapy, 2 days for encorafenib combined with binimetinib, and 12 days for vemurafenib [16].

However, these recommendations are largely based on toxicity reports involving RT administered with BRAFi or MEKi monotherapy [17–23]. More recent studies have suggested that BRAFi/MEKi combination may, in fact, be safely delivered concurrently with RT with minimal interruption to avoid disease progression [24–27], although larger prospective trials are lacking. Recently published framework by the European Society for Medical Oncology (ESMO) and the European Society for Radiotherapy and Oncology (ESTRO) claim that RT and concurrent proliferation inhibitors may impair normal-tissue reparation and thereby increase acute toxicity, especially in rapidly regenerating tissues. The mixed results indicate that further research is needed [28].

This retrospective study aims to evaluate the efficacy and safety of RT in patients progressing on BRAFi/MEKi therapy, with particular focus on survival outcomes in relation to subsequent systemic treatment strategies.

## Patients/material and methods

### Study population

This retrospective, population-based study included all patients with advanced melanoma who received RT for disease progression on TT in Stockholm (~2.5 million inhabitants), between 2015 and 2023. Patients were identified using the ICD-10 diagnostic code for melanoma and the Swedish national procedure code for RT. Eligibility criteria were stage IV melanoma, any type of confirmed *BRAF* V600 mutation, ongoing treatment with BRAFi or BRAFi/MEKi, and RT received for radiological or clinical disease progression. The study is reported in accordance with the STROBE guidelines. Baseline characteristics were assessed at the time of RT initiation. Data on treatment details, dosimetry parameters, and survival outcomes were collected from medical records. Information on RT-related adverse events (AEs) was obtained from patients’ charts and reviewed by the study authors. New or worsening AEs occurring up to 3 months post RT were graded according to Common Terminology Criteria for Adverse Events version 5.0 (CTCAE v5.0).

Oligometastatic disease was defined as one to five metastases as defined by ESTRO and ASTRO (American Society for Radiation Oncology) consensus [29]. Only the RT session delivered at first progression for each patient was analyzed. Equivalent dose in 2 Gy fractions (EQD2) was calculated assuming a normal tissue α/β ratio of 10. Patients were classified as having derived benefit from RT if they achieved a radiological complete or partial response. Patients with stable disease, or those without radiological follow-up, were only considered to have benefited if they had documented symptom relief. Response evaluation was based on the study authors assessment of radiological or clinical reports. Radiological assessment was performed according to clinical routine, typically every 3 months. Temporary treatment interruption was defined as at least 2 days of interruption with resumed TT post-RT. Patients were grouped according to the management of systemic therapy before, during, and after RT:

RT-STOP: patients who discontinued TT up to 1 month of initiating RT and did not commence subsequent TT or ICI treatment.

RT-TT: patients who continued TT for at least 1 month following the initiation of RT.

RT-ICI: patients who discontinued TT up to 1 month of initiating RT and started ICI therapy within 3 months.

### Statistics

Study endpoints were overall survival (OS), PFS, local efficacy, and RT-related toxicity. For descriptive data, median value and range was used for continuous variables, while counts and percentages were used for categorical variables. PFS was calculated from the start of RT to radiological or clinical disease progression (enlargement of existing metastases and/or development of new metastatic lesions) or death from any cause, whichever occurred first. Patients without progression or death were censored at the date of last clinical or radiological follow-up. OS was measured from the timepoint of RT to death from any cause or the date of last follow-up if alive. Survival curves were generated using the Kaplan–Meier method, and differences between cohorts were assessed with the log-rank test. The Cox proportional hazards model was used to determine significant prognostic factors of OS and to estimate hazard ratios (HRs) with associated 95% confidence intervals (CI). Missing data were handled using a complete case analysis approach. Differences in proportions of AEs between groups were assessed using Fisher’s exact test. *P*-values < 0.05 were considered statistically significant. All statistical analyses were conducted using STATISTICA (Dell Inc. (2016)), version 13.

## Results

### Patient characteristics

**Figure 1:**
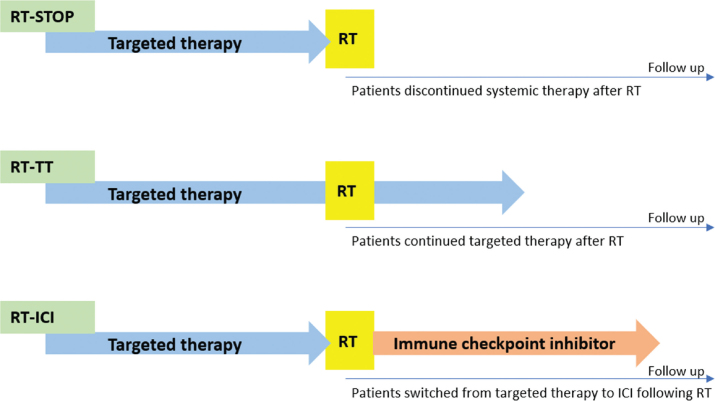
Patient stratification according to systemic treatment following radiotherapy (RT). Patients were categorized into three cohorts based on systemic therapy approach: RT-STOP: patients discontinuing targeted therapy (TT) after RT. RT-TT, patients continuing TT after RT. RT-ICI, patients transitioning to immune checkpoint inhibitor (ICI) after RT.

The analysis included 63 patients, with a median follow-up of 69.1, 65.0, and 87.2 months in RT-STOP, RT-TT, and RT-ICI, respectively. Follow-up was complete for the majority of patients, at data cut-off, 59/63 patients had died and 4 were alive and censored. All patients had a *BRAF* V600 mutation, however, the specific V600 subtype was recorded when available, but was not specified in the majority of pathology reports. Twenty-two patients were assigned to the RT-STOP group, 26 patients to the RT-TT group, and 15 to the RT-ICI group (Figure 1). The median age was 55 years (range, 28–88 years), and the cohort comprised approximately equal distribution of male and female patients (Table 1). Eastern Cooperative Oncology Group (ECOG) performance status differed numerically between cohorts. In the RT-STOP cohort, 13.6% had ECOG 0, 45.5% ECOG 1, and 40.9% ECOG 2–3. In the RT-TT cohort, the corresponding proportions were 46.2%, 46.2%, and 7.7%, respectively, and in the RT-ICI cohort 40.0%, 40.0%, and 20.0%. An elevated lactate dehydrogenase (LDH) level was observed in most patients (89%). M stage differed between cohorts. In the RT-STOP cohort, 4.5% had M1a, 0.0% M1b, 45.5% M1c, and 50.0% M1d. In the RT-TT cohort the corresponding proportions were 7.7%, 15.4%, 30.8% and, 46.2% respectively, and in the RT-ICI cohort 20.0%, 0.0%, 60.0% and 20.0%. A higher proportion of patients presented with polymetastatic disease compared with oligometastatic disease (76.2% vs. 23.8%).

**Table 1:** Metastatic melanoma patients treated with radiotherapy when progressing on BRAFi/MEKi.

Characteristic	Total *n* = 63 (%)	RT-STOP *n* = 22 (%)	RT-TT *n* = 26 (%)	RT-ICI *n* = 15 (%)
Age, median (range)	55 (28–88)	64 (28–88)	62 (28–88)	51 (34–82)
Sex, *n* (%)				
Female	32 (50.8)	11 (50.0)	14 (53.8)	7 (46.7)
Male	31 (49.2)	11 (50.0)	12 (46.2)	8 (53.3)
ECOG performance status, *n* (%)				
0	21 (33.3)	3 (13.6)	12 (46.2)	6 (40.0)
1	28 (44.4)	10 (45.5)	12 (46.2)	6 (40.0)
2–3	14 (22.2)	9 (40.9)	2 (7.7)	3 (20.0)
LDH level, *n* (%)				
≤ ULN	6 (9.7)	3 (13.6)	0 (0.0)	3 (20.0)
> ULN	56 (90.3)	19 (86.4)	25 (100.0)	12 (80.0)
Missing	1	0	1	0
M stage, *n* (%)				
M1a	6 (9.5)	1 (4.5)	2 (7.7)	3 (20.0)
M1b	4 (6.3)	0 (0.0)	4 (15.4)	0 (0.0)
M1c	27 (42.9)	10 (45.5)	8 (30.8)	9 (60.0)
M1d	26 (41.3)	11 (50.0)	12 (46.2)	3 (20.0)
Tumor burden, *n* (%)				
Oligometastatic disease	15 (23.8)	6 (27.3)	7 (26.9)	2 (13.3)
Polymetastatic disease	48 (76.2)	16 (72.7)	19 (73.1)	13 (86.7)
Previous RT, *n* (%)				
Yes	18 (28.6)	7 (31.8)	7 (26.9)	4 (26.7)
No	45 (71.4)	15 (68.2)	19 (73.1)	11 (73.3)
RT to all metastatic lesions, *n* (%)				
Yes	4 (6.3)	2 (9.1)	1 (3.8)	1 (6.7)
No	59 (93.4)	20 (90.9)	25 (96.2)	14 (93.3)
Number of RT targets, *n* (%)				
1	59 (93.4)	19 (86.4)	26 (100.0)	14 (93.3)
2	4 (6.3)	3 (13.6)	0 (0.0)	1 (6.7)
RT target organ, *n* (%)				
Skin/soft tissue	11 (17.5)	3 (13.6)	4 (15.4)	4 (26.7)
Lymph nodes	9 (14.3)	3 (13.6)	3 (11.5)	3 (20.0)
Lung/mediastinum	8 (12.7)	1 (4.5)	6 (23.1)	1 (6.7)
Abdominal	4 (6.3)	1 (4.5)	2 (7.7)	1 (6.7)
Skeletal	17 (27.0)	8 (36.4)	5 (19.2)	4 (26.7)
CNS	14 (22.2)	6 (27.3)	6 (23.1)	2 (13.3)
Type of concurrent targeted therapy, *n* (%)				
Encorafenib + binimetinib	16 (25.4)	7 (31.8)	7 (26.9)	2 (13.3)
Dabrafenib + trametinib	41 (65.1)	12 (54.5)	17 (65.4)	12 (80.0)
Vemurafenib + cobimetinib	2 (3.2)	2 (9.1)	0 (0.0)	0 (0.0)
Vemurafenib + erlotinib	1 (1.6)	0 (0.0)	0 (0.0)	1 (6.7)
Vemurafenib + trametinib	1 (1.6)	1 (4.5)	0 (0.0)	0 (0.0)
Vemurafenib	1 (1.6)	0 (0.0)	1 (3.8)	0 (0.0)
Dabrafenib	1 (1.6)	0 (0.0)	1 (3.8)	0 (0.0)
Median duration of treatment with TT, months (range)	8 (1–41)	7.5 (2–41)	10 (1–38)	6 (2–22)
BRAF/MEKi interruption during RT, *n* (%)				
No interruption	11 (17.5)	4 (18.2)	7 (26.9)	0 (0.0)
Treatment interruption (permanent or temporary)	52 (82.5)	18 (81.8)	19 (73.1)	15 (100.0)
Median temporary interruption time, days (range)	13 (3–23)	19 (3–21)	13 (4–23)	8 (5–11)
Number of previous systemic treatments, *n* (%)				
0	36 (57.1)	14 (63.6)	11 (42.3)	11 (73.3)
1	22 (34.9)	5 (22.7)	13 (50.0)	4 (26.7)
2–3	5 (7.9)	3 (13.6)	2 (7.7)	0 (0.0)
ICI prior to TT, *n* (%)				
No prior ICI	36 (57.1)	13 (59.1)	12 (46.2)	11 (73.3)
Anti-CTLA-4 + anti-PD1	9 (14.3)	4 (18.2)	5 (19.2)	0 (0.0)
Anti-PD1	18 (28.6)	5 (22.7)	9 (34.6)	4 (26.7)

BRAFi: BRAF inhibitor; MEKi: MEK inhibitor; RT: radiotherapy; TT: targeted therapy; ICI: immune checkpoint inhibitors; LDH: lactate dehydrogenase; CNS: central nervous system; ULN: upper limit normal; CTLA-4: cytotoxic T-lymphocyte associated protein 4; PD1: Programmed cell death protein 1

### Radiotherapy and systemic treatment

Approximately two-thirds of patients had not received prior RT. Only four cases (6.3%) received RT to all metastatic lesions. In most cases (92.1%), RT was directed at a single metastatic site. The skeleton was the most frequently irradiated organ (27.0%), followed by the central nervous system (CNS) (22.2%), skin and soft tissue (15.9%), lymph node (15.9%), lung (11.1%), and abdominal lesions (6.3%). Fifty-five patients (87.3%) received 3D conformal RT and 8 (12.7%) received stereotactic radiation therapy. The three most commonly prescribed doses were 4 Gy x5 (27%), 8 Gy x2 (27%), and 6 Gy x5 (14%) (Supplementary Table 1). In the RT-STOP, RT-TT, and RT-ICI groups, 27.3%, 42.3%, and 13.3% of patients, respectively, received RT for a single progressing metastatic lesion (Supplementary Table 2). The primary indication for RT was pain palliation in 27.3%, 23.1%, and 46.7% of patients in the respective groups, whereas RT was administered for oligoprogression in 27.3%, 38.5%, and 13.3%, respectively

The two most used BRAFi/MEKi were dabrafenib plus trametinib (65.1%) and encorafenib plus binimetinib (25.4%). The median duration of TT was 8 months (range, 1–41 months), calculated from the initiation to the discontinuation of TT. Eleven patients (17.5%) had no interruption of TT during RT, 25 (39.7%) had a temporary interruption, and 27 (42.9%) had a permanent cessation. The median time of temporary treatment interruption was 13 days (range 3–23 days). Thirty-six (57.1%) patients were treatment naïve when starting TT, while 22 (34.9%) had received one prior systemic treatment and 5 (7.9%) had received two or three. The most common prior systemic treatment was anti-PD1 therapy (28.6%) followed by combination anti-cytotoxic T-Lymphocyte Associated Protein 4 (CTLA-4) inhibitor and anti-programmed cell death protein 1 (PD-1) inhibitor (14.3%) (Table 1). Patients were treated with TT as first line systemic treatment in 36 patients and the three most common reasons were symptomatic disease, high tumor burden, or poor ECOG performance status (61.1%), initiation of TT before the approval of anti–PD-1 therapy (19.4%), and the presence of autoimmune disease (11.1%).

### Adverse events

Twenty-four out of 63 patients (38.1%) experienced RT-related AEs (Table 2). The most frequent were fatigue (*n* = 9), pain (*n* = 7), and nausea (*n* = 4) (Supplementary Table 3). No grade ≥ 3 AEs were observed. Among patients who continued TT without interruption during RT (*n* = 11), 6 patients (54.5%) experienced RT-related AEs (Supplementary Table 4), compared with 18 (34.6%) of those who interrupted TT during RT (*p* = 0.307). There was a statistically significant difference in RT-related AEs between patients treated with dabrafenib (28.6%) compared to encorafenib (62.5%) (*p* = 0.032). When stratified by treatment interruption, the highest proportion of AEs was observed among encorafenib-treated patients who underwent treatment interruption (70.0%), which was significantly higher than among dabrafenib-treated patients (24.3%) (*p* = 0.020) (Table 2). Seventeen (35.4%) of 48 patients who received EQD2 < 40 Gy and 8 (53.3%) of 15 patients who received EQD2 ≥ 40 Gy experienced RT-related AEs (*p* = 0.241).

**Table 2:** Radiotherapy-related adverse events in patients receiving RT when progressing on targeted therapy.*

Adverse events	All patients (*n* = 63)	Vemurafenib based therapy (*n* = 5)	Dabrafenib based therapy (*n* = 42)	Encorafenib based therapy (*n* = 16)	*P*-value Vem vs. Dabra	*P*-value Vem vs. Enco	*P*-value Dabra vs. Enco
Any AEs, *n* (%)	24 (38.1%)	2 (40.0%)	12 (28.6%)	10 (62.5%)	0.627	0.611	0.032
Fatigue, *n*	9	0	4	5			
Skin, *n*	7	0	5	2			
Gastrointestinal, *n*	6	1	2	3			
Central nervous system, *n*	6	0	4	2			
Respiratory, *n*	1	0	1	0			
Pain, *n*	7	1	3	3			

	Continued daily intake TT throughout RT:						
	All continuing TT (*n* = 11)	Vemurafenib based therapy (*n* = 0)	Dabrafenib based therapy (*n* = 5)	Encorafenib based therapy (*n* = 6)			

Any AEs, *n* (%)	6 (54.5%)	NA	3 (60.0%)	3 (50.0%)	NA	NA	1
Fatigue, *n*	3	NA	1	2			
Skin, *n*	4	NA	3	1			
Gastrointestinal, *n*	1	NA	1	0			
Central nervous system, *n*	0	NA	0	0			
Respiratory, *n*	1	NA	1	0			
Pain, *n*	1	NA	0	1			

	Discontinued intake TT during RT:						
	All discontinuing TT (*n* = 52)	Vemurafenib based therapy (*n* = 5)	Dabrafenib based therapy (*n* = 37)	Encorafenib based therapy (*n* = 10)			

Any AEs, *n* (%)	18 (34.6%)	2 (40.0%)	9 (24.3%)	7 (70.0%)	0.593	0.153	0.020
Fatigue, *n*	6	0	3	3			
Skin, *n*	3	0	2	1			
Gastrointestinal, *n*	5	1	1	3			
Central nervous system, *n*	6	0	4	2			
Respiratory, *n*	0	0	0	0			
Pain, *n*	6	1	3	2			
*P*-value continued vs. discontinued TT	0.307	NA	0.131	0.607			

RT: radiotherapy; TT: targeted therapy; AE: adverse events.

* All AEs were grade 1–2 (no ≥3 AEs). AEs were assessed according to Common Terminology Criteria for Adverse Events version 5.0 (CTCAE v5.0) and grouped by organ system for readability, reflecting common clinical categorization used in oncologic practice.

### Efficacy

**Figure 2:**
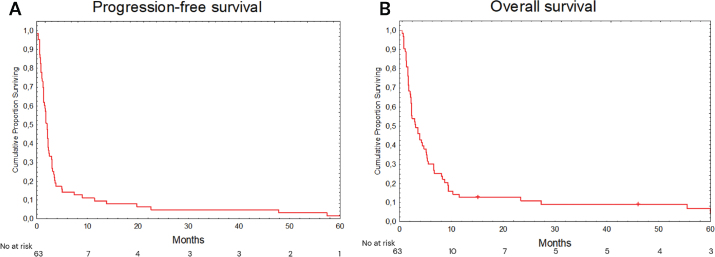
Kaplan–Meier curves of (A) progression-free survival (PFS) and (B) overall survival (OS) in patients with metastatic melanoma receiving radiotherapy (RT) at the time of disease progression on targeted therapy (TT).

**Figure 3:**
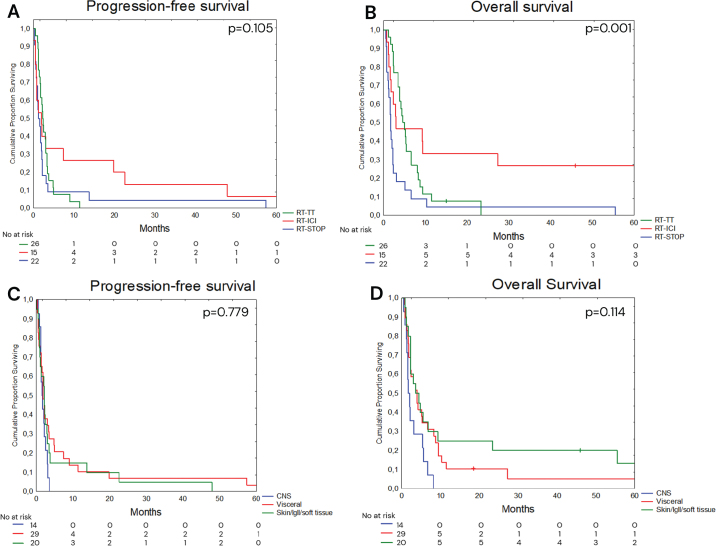
Kaplan–Meier curves of progression-free survival (PFS) and overall survival (OS) in patients with metastatic melanoma receiving radiotherapy (RT) at the time of disease progression on targeted therapy (TT). (A) PFS according to systemic management: RT-STOP (patients discontinuing TT), RT-TT (patients continuing TT after RT), and RT-ICI (patients transitioning from TT to immune checkpoint inhibitor (ICI) therapy after RT). (B) OS in the RT-STOP, RT-TT, and RT-ICI cohorts. (C) PFS according to the irradiated site: central nervous system (CNS), visceral organs, or skin/lymph nodes/soft tissue. (D) OS according to the irradiated site: CNS, visceral organs, or skin/lymph nodes/soft tissue.

Information on radiological response or symptom relief in the irradiated region following completion of RT was available for 55 patients, of whom 28 patients (50.9%) were deemed to have derived a local benefit from RT (defined as radiological complete or partial response, or documented symptom relief in the absence of objective response). In RT-STOP, RT-TT, and RT-ICI the proportion that derived benefit was 35.3%, 56.0%, and 61.5%, respectively. In the whole cohort, median PFS and OS were 1.9 (CI 95% 1.6–2.3) and 3.1 (CI 95% 2.8–3.3) months, respectively (Figure 2A and 2B). One-year OS was 0.0% in patients receiving irradiation to the CNS, 10.2% to visceral organs, and 24.9% for skin, soft tissue or lymph nodes (*p* = 0.114). Using skin, soft tissue or lymph node as reference, CNS demonstrated inferior OS (HR 2.59, 95% CI 1.23–5.45), *p* = 0.012) while visceral organs demonstrated no significant differences (HR 1.33, 95% CI 0.72–2.46, *p* = 0.356) (Figure 3C and 3D).

**Figure 4:**
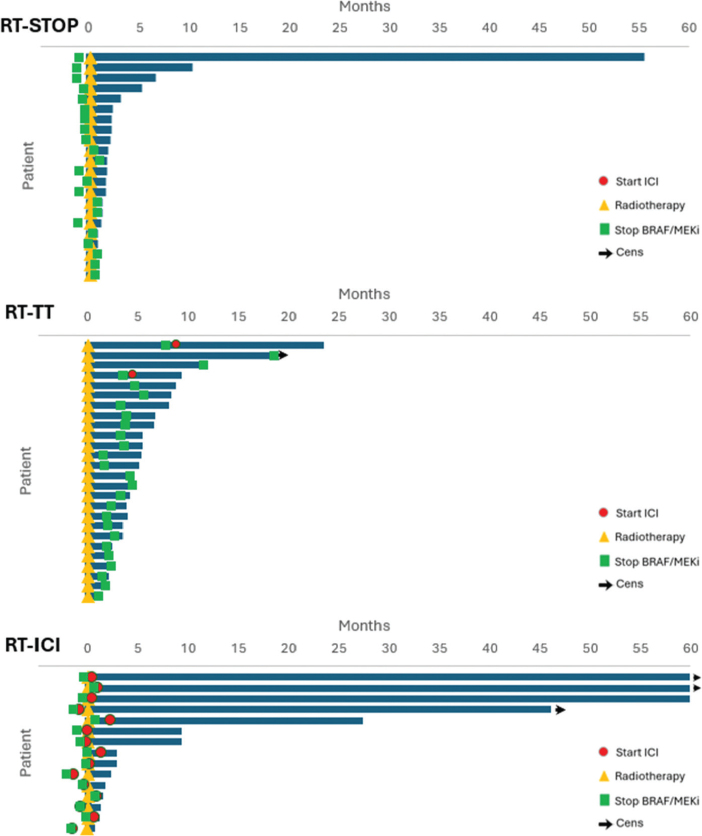
Swimmer plots of patients with metastatic melanoma receiving radiotherapy (RT) at the time of disease progression on targeted therapy (TT). Patients were categorized based on systemic therapy approach: RT-STOP (patients discontinuing TT), RT-TT (patients continuing TT after RT), and RT-ICI (patients transitioning from TT to immune checkpoint inhibitor (ICI) therapy after RT).

Six-month PFS rates were 9.1%, 7.6%, and 22.4% in RT-STOP, RT-TT, and RT-ICI, respectively (*p* = 0.105). The 6- and 12-month OS rates in RT-STOP were 13.6% and 4.9%, in RT-TT 34.6% and 7.6%, and in RT-ICI 46.8% and 33.4% (*p* = 0.001). Using RT-STOP as the reference group, both RT-TT (HR 0.46, 95% CI 0.26–0.84, *p* = 0.011) and RT-ICI (HR 0.39, 95% CI 0.18–0.82, *p* = 0.013) were associated with improved OS (Figure 3A and 3B). When adjusting for stage (M1c–M1d vs. M1a–M1b), ECOG (2–3 vs. 0–1) and LDH level (elevated vs. non-elevated), the HR for OS was 0.40 (95% CI 0.15–0.91) in RT-ICI compared to RT-STOP and 0.51 (95% CI 0.27–0.96) in RT-TT compared to RT-STOP. A swimmer plot demonstrating individual patient survival including the timing of systemic treatments across the three cohorts: RT-STOP, RT-TT, and RT-ICI, illustrating a median OS of 1.7 (95% CI 1.2–2.2), 4.7 (95% CI 3.9–6.7), and 3.0 (95% 1.8–9.3) months, respectively (Figure 4).

## Discussion and conclusion

In this study, we investigated management strategies upon disease progression during BRAFi/MEKi treatment. Our findings demonstrate that RT is a safe option in this context, from which a considerable proportion of patients appear to obtain local benefit, and that continuing TT beyond progression or switching to ICI is associated with improved OS compared to discontinuation of TT. However, the study also points to the poor survival outcomes in this situation, with a median PFS and OS of 1.9 and 3.1 months. While treatment with BRAFi/MEKi in metastatic melanoma typically yields a PFS of 11–15 months and an OS of 22 months [7, 10], outcomes following progression on TT have been reported to be poorer [11, 15, 30, 31]. However, data specifically addressing systemic treatment strategies in patients with progression treated with RT remain limited.

Our study suggests that patients who continued TT beyond progression had improved OS compared with patients term-inating TT. This finding aligns with the phase 1 trial by Kim et al. in which 18 patients who continued vemurafenib for more than 30 days after receiving local therapy at a site of disease progression had improved PFS and OS compared with those who discontinued vemurafenib [32]. Additionally, a retro-spective case series describes five patients who achieved prolonged benefit from dabrafenib and trametinib when receiving RT while continuing TT beyond progression [27]. When comparing strategies with systemic treatment, we observed that the median OS appeared longer in the RT-TT group (4.7 months) than in the RT-ICI group (3.0 months), yet the 1-year OS rate favored RT-ICI (7.6% in RT-TT, and 33.4% in RT-ICI). This may suggest that continuing TT beyond progression after RT provides shorter survival extension for most patients, with long-term benefit generally not expected. In contrast, switching to ICI after RT carries substantial risk, as most patients do not respond, although a subset may achieve durable long-term survival, as observed in the SECOMBIT and DREAMseq trials [11, 15]. Consequently, we believe that deciding between these two treatment strategies should involve careful multidisciplinary evaluation and patient-informed decision-making. However, these findings should be interpreted cautiously given the small subgroup sizes, wide CIs, and the fact that the proportional hazard assumption was not formally tested. Moreover, PFS was not significant whereas OS was, and both endpoints were calculated from the time of RT following progression on TT, thus primarily reflecting post-progression survival. As most patients received single-site RT while continuing systemic therapy, the observed survival differences likely reflect underlying disease biology and systemic treatment effects rather than the independent effect of RT. A plausible explanation for the improved OS observed in RT-TT and RT-ICI compared with RT-STOP is related to confounding by indication: patients in the RT-STOP group may have had clinical characteristics suggesting they were too unwell to continue treatment. We observed numerical differences in the indications for RT between the treatment groups. In the RT-TT cohort, RT was more frequently administered for oligoprogression (38.5%), suggesting that local therapy was used to control limited disease progression while maintaining systemic treatment. In contrast, RT in the RT-ICI group was more often delivered for pain palliation (46.7%), which may reflect a higher burden of symptomatic disease. In the RT-STOP groups, indications were more evenly distributed between pain palliation, neurologic indications, and oligoprogression (27.3% each), suggesting a more heterogeneous population. These differences in treatment indication may represent variations in clinical management strategies and could have influenced outcomes. However, both RT-TT and RT-ICI remained significantly associated with improved OS compared to RT-STOP after adjustment for M stage, LDH level, and ECOG performance status, suggesting further prospective studies are warranted to explore this hypothesis.

Two aspects of interest warrant discussion. Firstly, it would have been informative to investigate the distribution of *BRAF* mutation subtypes (V600E, V600K, and others) between our three cohorts. Previous studies have reported differences in survival and treatment response between specific subtypes. Patients with V600E mutations generally show longer PFS with TT, whereas those with V600K mutations often have a higher tumor mutational burden and respond better to ICI [1]. Since the most frequently used testing method in our cohort did not distinguish between these subtypes, this may represent a limitation of the study. However, these observations largely derive from first-line treatment settings, and their relevance in the post-progression setting evaluated in our cohort remains unclear. Secondly, a potential synergistic effect between RT and ICI is of interest, particularly given the limited efficacy of second-line ICI after BRAFi/MEKi progression. However, this study was not designed to evaluate treatment response rates or treatment synergy. Further studies are warranted to explore this potential interaction.

In the overall cohort, 50.9% of patients derived clinical benefit from RT. Numerically, the proportion was lower in the RT-STOP group (35.3%) compared with the RT-TT (56.0%) and RT-ICI (61.5%) groups. Several factors may explain this difference. Patients in the RT-STOP group may have had poorer clinical status or more advanced disease, which may have reduced the likelihood of deriving benefit from RT. In addition, less frequent follow-up and shorter survival in the RT-STOP group may have limited the detection of RT-related benefit. In such cases, the potential benefits of RT should be carefully weighed against the burden of the treatment, including time spent receiving care, and discussed with patients in the context of palliative treatment goals. Although the higher proportion of benefit observed in the RT-ICI group raises the possibility that RT may be particularly effective when administered alongside ICI, consistent with our earlier observation [33]. However, the results were similar to those in the RT-TT group, preventing conclusions regarding a specific RT–ICI interaction.

Preclinical studies [17, 23, 34] suggested that BRAFi could enhance the antitumor effect of RT by acting as radiosensitizers, but clinical evidence supporting a clear therapeutic advantage of this combination remains limited. However, most clinical studies have assessed RT in settings other than post-progression, such as during response or in combination with upfront TT. Two retrospective studies have examined the impact of adding BRAFi to stereotactic radiosurgery (SRS) for brain metastases with conflicting results for local control [35, 36], and neither demonstrated improvements in OS. Furthermore, the phase 2 EBRAIN/GEM-1809 trial found no additional benefit in local control when RT was added to patients responding to encorafenib plus binimetinib [37]. Likewise, a systematic review reported comparable outcomes between TT combined with SRS and dabrafenib with trametinib alone [38].

There is a concern regarding potential toxicity when combining RT with TT. Conversely, there is also apprehension about the risk of tumor progression during treatment interruptions of TT. In this study, we observed no grade ≥ 3 AEs, which supports the safety profile of these combined modalities. However, most patients temporarily interrupted TT (median 13 days, range 3–23). When comparing patients who continued TT with those who paused treatment during RT, a numerical increase in toxicity was observed (54.5 vs. 34.6%). However, the sample was small (*n* = 11), and the difference did not reach statistical significance. A similar pattern was observed between targeted agents, with increased toxicity among patients treated with encorafenib-based therapy compared with dabrafenib-based therapy (62.5 vs 28.6%) (*p* = 0.032). Notably, encorafenib has a high affinity for BRAF and a prolonged dissociation half-life [39], which may sustain target inhibition even during brief treatment interruptions and could theoretically enhance radiosensitization. Further investigation in larger cohorts is warranted to determine whether this observed difference in toxicity is clinically meaningful.

Several studies support the safety of combining RT with TT. Preclinical data demonstrate biological compatibility, with trametinib enhancing RT effects without added toxicity [23]. Clinical case reports likewise show minimal RT-related AEs when TT is continued during RT [24, 27]. Similarly, a retrospective study including 32 patients treated with either stereotactic or conventional RT to both intracranial and extracranial sites reported no difference in toxicity whether RT was delivered prior to TT, during TT, or without interruption [26]. Importantly, prospective trials now support these observations: the EBRAIN/GEM-1809 phase II trial demonstrated that encorafenib and binimetinib could be safely adminstered with radiosurgery, stereotactic RT, or whole brain RT in patients with intracranial metastases, using a 24-hour interruption prior to and after RT [37]. Furthermore, a phase I/II trial found low rates of grade 1–2 toxicity during uninterrupted dabrafenib and trametinib with palliative RT to non-visceral extracranial sites [25].

In contrast, some studies have reported increased toxicity with the combination of RT and TT. A case report descibed a patient treated with dabrafenib and tremetinib and developed a severe radiation recall dermatitis following RT [40]. This observation is further supported by a retrospective study by Hecht et al. [17], which reported toxicity in 57% of RT courses administered with concurrent BRAFi therapy. Acute grade ≥ 2 radiodermatitis occurred in 36% of patients and was more common with vemurafenib (40%) than dabrafenib (26%). The authors attributed this difference either to an immunologic enhancement or to vemurafenib’s broader kinase activity. Similarly, a prospective phase I trial reported significant toxicity, with a maximum tolerated dose < 1.5 mg in patients treated with concurrent trametinib and WBRT [21]. According to the ECOG consensus from 2016, BRAFi and MEKi should be withheld for at least 3 days before and after fractionated RT, and for at least 1 day before and after SRS. However, these guidelines are primarily based on BRAFi monotherapy, and there are no specific recommendations regarding encorafenib and binimetinib due to lack of data [41]. Our findings are broadly consistent with the latest recommendation from ESMO and ESTRO, which deem it necessary to individualize decisions regarding treatment interruption of TT, taking into account the irradiated tissue, RT dose and fractionation, and anticipated toxicity, rather than relying on fixed interruption intervals [28].

RT-related AEs may vary among different BRAFi/MEKi, but dosimetry also plays a role. The ECOG group recommended a dose per fraction < 4 Gy unless stereotactic techniques are used [41]. However, this recommendation is not practical in melanoma, a relatively radioresistant tumor to conventional fractionation [42]. In our cohort, all patients but one received dose fractions > 4 Gy, without any reported severe toxicity. Moreover, beyond dose per fraction, factors such as target location, treatment planning, and total dose also influence toxicity, as stated by the ESMO and ESTRO recommendation [28]. In our cohort, AE rates were not significantly different between those receiving EQD2 <40 Gy (35.4%) and those receiving EQD2 ≥ 40 Gy (53.3%). However, the higher-dose subgroup was small (*n* = 15), limiting statistical power. Consequently, a potential association between radiation dose and toxicity cannot be excluded and these findings should be interpreted with caution.

To our knowledge, this is the largest study specifically examining the role of RT in advanced melanoma patients failing TT, offering new insights into how RT fits within modern systemic treatments. While the retrospective, single-center design, a lack of comparator group without RT, absence of standardized follow-up, and modest cohort size are recognized limitations, the study provided real-world data in a clinical scenario that remains poorly defined.

To conclude, in patients with advanced melanoma progressing on TT, RT is a safe treatment option that provides a clinically relevant local benefit. For patients still eligible for ICI escalation, switching to dual ICI is usually the preferred systemic strategy. However, for those already resistant to prior ICI, treating progressing lesions with RT while continuing TT beyond progression offers evidence of survival benefit. Clinical decisions should be individualized, balancing potential benefits and risks, ideally discussed in multidisciplinary teams.

## Supplementary Material





## Data Availability

The data that support the findings of this study are available from the corresponding author E.H and specific data will be available on reasonable request.
