# Supplementary material for: The role of radiotherapy in patients with advanced melanoma failing targeted therapy
Source: Acta Oncol. 2026 Apr 24;65:45437. doi: 10.2340/ao.v65.45437 (PMC13127106; doi:10.2340/ao.v65.45437)
Supplement: Supplementary file 1 [file AO-65-45437-S1.pdf]

Supplementary material has been published as submitted. It has not been copyedited, or typeset by Acta Oncologica

Supplementary material

Supplementary Table 1. Dosimetric data				
	Total n=63 (%)	RT-STOP n=22 (%)	RT-TT n=26 (%)	RT-ICI n=15 (%)
Radiotherapy (RT) type, n (%)				
Stereotactic RT	8 (12.7)	1 (4.5)	6 (23.1)	1 (6.7)
3D-CRT*	55 (87.3)	21 (95.5)	20 (76.9)	14 (93.3)
Prescribed dose, n (%)				
15 Gy x3	3 (5.0)	0 (0.0)	3 (11.5)	0 (0.0)
10 Gy x5	2 (3.0)	0 (0.0)	2 (7.7)	0 (0.0)
8 Gy x5	1 (2.0)	0 (0.0)	0 (0.0)	1 (6.7)
8 Gy x2	17 (27.0)	8 (36.4)	6 (23.1)	3 (20.0)
8 Gy x1	8 (13.0)	2 (9.1)	3 (11.5)	3 (20.0)
6 Gy x5	9 (14.0)	3 (13.6)	3 (11.5)	3 (20.0)
5 Gy x5	3 (5.0)	1 (4.5)	1 (3.8)	1 (6.7)
4 Gy x5	17 (27.0)	7 (31.8)	6 (23.1)	4 (26.7)
4 Gy x4	1 (2.0)	0 (0.0)	1 (3.8)	0 (0.0)
4 Gy x3	1 (2.0)	1 (4.5)	0 (0.0)	0 (0.0)
3 Gy x10	1 (2.0)	0 (0.0)	1 (3.8)	0 (0.0)

*3D-Conformal Radiation Therapy

Supplementary Table 2. Clinical situations for receiving radiotherapy (RT) when progressing on targeted therapy (TT)				
	Total n=63 (%)	RT-STOP n=22 (%)	RT-TT n=26 (%)	RT-ICI n=15 (%)
Total number of progressing lesions, n (%)				
0-1	19 (30.2)	6 (27.3)	11 (42.3)	2 (13.3)
2-3	15 (23.8)	5 (22.7)	7 (26.9)	3 (20.0)
4-5	8 (12.7)	4 (18.2)	1 (3.8)	3 (20.0)
6-10	12 (19.0)	3 (13.6)	4 (15.4)	5 (33.3)
>10	9 (14.3)	4 (18.2)	3 (11.5)	2 (13.3)
Indication for RT, n (%)				
Pain palliation	19 (30.2)	6 (27.3)	6 (23.1)	7 (46.7)
Oligoprogression	18 (28.6)	6 (27.3)	10 (38.5)	2 (13.3)
Visceral Organ Compression	6 (9.5)	2 (9.1)	2 (7.7)	2 (13.3)
CNS and Neurologic Indications	14 (22.2)	6 (27.3)	5 (19.2)	3 (20.0)
Bleeding / Ulcerative Lesions	6 (9.5)	2 (9.1)	3 (11.5)	1 (6.7)

TT: targeted therapy
ICI: immune checkpoint inhibitors

Supplementary Table 3. Grade 1-2 radiotherapy-related adverse events (n=63)	
Any AEs, n (%)	24 (38.1%)
Skin, n (%)	
Redness	2 (3.2)
Hyperpigmentation	1 (1.6)
Edema	1 (1.6)
Desquamation	1 (1.6)
Pruritus	2 (3.2)
Gastrointestinal, n (%)	
Nausea	4 (6.3)
Abdominal pain	2 (3.2)
Central nervous system, n (%)	
Headache	1 (1.6)
Paresthesia	1 (1.6)
Dizziness	2 (3.2)
Amnesia	2 (3.2)
Respiratory, n (%)	
Cough	1 (1.6)
Pain, n (%)	
Pain	7 (11.1)
Fatigue, n (%)	
Fatigue	9 (14.3)

Supplementary Table 4. Patient continuing targeted therapy without interruption during radiotherapy					
Patient	Cohort	Radiotherapy target	Fractionation scheme	BRAFi/MEKi	Radiotherapy-related adverse events
1	RT-STOP	Mediastinal lymph node	4 Gy x5	Encorafenib + binimetinib	Fatigue grade 1
2	RT-TT	Lung	8 Gy x2	Dabrafenib + trametinib	Cough grade 1. Nausea grade 1.
3	RT-TT	Skin	8 Gy x2	Dabrafenib	Pruritus grade 1. Hyperpigmentation grade 2. Desquamation grade 1.
4	RT-TT	Axillary lymph node	6 Gy x5	Encorafenib + binimetinib	Pain grade 1. Edema grade 1.
5	RT-STOP	Lung	8 Gy x2	Dabrafenib + trametinib	0
6	RT-STOP	Skeletal	8 Gy x1	Encorafenib + binimetinib	0
7	RT-TT	Skeletal	8 Gy x2	Encorafenib + binimetinib	0
8	RT-TT	Axillary lymph node	6 Gy x5	Dabrafenib + trametinib	Fatigue grade 1
9	RT-TT	Brain	4 Gy x5	Encorafenib + binimetinib	Fatigue grade 1
10	RT-TT	Brain	6 Gy x5	Dabrafenib + trametinib	0
11	RT-STOP	Skeletal	8 Gy x2	Encorafenib + binimetinib	0
